# Supplementary material for: Similar but different: Profiling secondary school students based on their perceived motivational climate and psychological need-based experiences in physical education
Source: PLoS One. 2020 Feb 10;15(2):e0228859. doi: 10.1371/journal.pone.0228859 (PMC7010287; doi:10.1371/journal.pone.0228859)
Supplement: Attachment — Submitted filename: Response to Reviewers 1.docx [file pone.0228859.s002.docx]

Response to Reviewers

**Comments academic editor**

**1. *I truly enjoyed reading this manuscript on profiling secondary students in***

***physical education based on motivation and psychological needs. It was***

***well-written and easy to read and follow. The methods appear to be sound,***

***and the data analyses are solid. The discussion is well-organized and***

***explains each of the research questions and elaborates on the results. While***

***motivation in physical education is of very high interest to me, I am not an***

***expert in the area. However, I do believe most of the appropriate literature***

***has been referenced.***

Thank you for your compliments and clear comments**.**

**2. *Lines 106 and 110 are incomplete sentences.***

We adjusted this by adding commas that link the sentences as intended.

Page 4, lines 113-120 (in revised manuscript with track changes):

‘A mastery climate is characterized as an environment in which students perceive they

are rewarded for effort, personal development, learning, cooperation and individual

improvement, whereas a performance climate promotes and facilitates social comparison

between students by rewarding superior performance [5]. Overall, perceived mastery

climates are positively associated with several adaptive constructs, such as higher levels

of self-determined regulation, greater satisfaction of the basic psychological needs and

greater enjoyment [27-29], while perceptions of a performance climate have been linked

to less desirable outcomes, such as boredom, anxiety and antisocial moral attitudes

[27,30].’

**3. *Is there any data to determine if the classes/teachers are, in fact, are***

***mastery or performance climate? Or is this all based on the students’***

***perceptions? Please clarify.***

In this study there is no data to determine whether the PE classes were mastery or

performance climate. The motivational profiles are solely defined based on the students’

perceptions only. Some examples where this is indicated in the paper are:

• The title:

‘Similar but different: Profiling Secondary School Students based on their Perceived

Motivational Climate and Psychological Need-Based Experiences in Physical

Education’

• Page 3, line 87-93 (in revised manuscript with track changes):

‘The purpose of this paper is therefore to gain more insight into students’ perception

of the motivational learning climate in secondary school PE in the Netherlands. We

explore whether different constructs of student motivation in the context of PE (i.e.

perceived psychological need satisfaction and need frustration, and perceived

teacher-initiated mastery climate versus performance climate) combine into distinct

student profiles, aiming to unveil motivational similarities and differences between

students’ experiences in PE.’

• Page 5, line 183-185 (in revised manuscript with track changes):

‘The present study aims to expand on the current profiling research, by exploring how

students perceive the learning environment in PE by combining the motivational

frameworks of AGT and SDT.’

Previous research did use observational methods to determine the class climate (e.g.

Haerens et al, 2013; Morgan, Sproule, Weigand & Carpenter, 2005), and such research

is underway in our research group as well. We believe that this could indeed have been a

valuable addition to our study. Unfortunately, this was not feasible due to time constraints.

In line with your comment, the abstract of our paper may not sufficiently state that it only

pertains to the perception of students. Therefore, we made these changes to the abstract:

Page 2, line 44-59 (in revised manuscript with track changes):

‘The purpose of this study was to provide more insight into how the physical education

(PE) context can be better tailored to the diverse motivational demands of secondary

school students. Therefore, we examined how different constructs of student motivation in

the context of PE combine into distinct motivational profiles, aiming to unveil motivational

similarities and differences between students’ PE experiences. Participants were 2,562

Dutch secondary school students, aged 12-18, from 24 different schools. Students

responded to questionnaires assessing their perception of psychological need satisfaction

and frustration, and perceived mastery and performance climate in PE. In order to

interpret the emerging profiles additional variables were assessed (i.e. demographic,

motivational and PE-related variables). Two-step cluster analysis identified three

meaningful profiles labelled as *negative perceivers*, *moderate perceivers* and *positive*

*perceivers*. These three profiles differed significantly with regard to perceived

psychological need satisfaction and frustration and their perception of the motivational

climate. This study demonstrates that students can be grouped in distinct profiles based

on their perceptions of the motivational PE environment. Consequently, the insights

obtained could assist PE teachers in designing instructional strategies that target

students’ differential motivational needs.’

In the Limitations we also added:

Page 15, line 522-533 (in revised manuscript with track changes):

‘Since our study employed a cross-sectional design, causality cannot be inferred. In order

to conclude with any certainty whether a desirable motivational climate is responsible for

changing the quality of motivation of students, valuation of PE or even leisure time sport

participation, intervention research is needed. Moreover, in this study the motivational PE

climate was determined by the perceptions of students only. There is no data on which it

can be concluded whether the PE classes were, in fact, mastery or performance

orientated or psychological need supportive or frustrating. We removed 268 students from

the sample due to missing data. It cannot be ruled out that these students were not

representative of the whole group. However, we expect the possible impact of this (with a

remaining N = 2562) to be within acceptable limits.

Furthermore, although this study provides a quantitative insight into students’ perceptions

of the motivational learning climate in Dutch secondary school PE, qualitative research is

needed to gain more detailed insight and understanding of students’ experiences and

needs in PE.’

***4. As a field, we encourage physical education teachers to employ a mastery***

***climate due to the seemingly negative impacts of a performance-related***

***climate. Are the results of this study suggesting that a performance climate***

***may be more beneficial for some students, depending on their profile?***

***Please elaborate.***

In this study we identified groups of students that differ in the way they perceive

secondary school PE in general. We also characterized these groups of students (i.e.

profiles) by gender, age, achievement goal orientation and motivational regulation.

Based on this study however, it cannot be determined if students in a given profile may

thrive more in a specific learning climate (i.e. performance or mastery climate) or benefit

more from, for example, providing choices during learning tasks (i.e. autonomy support)

than students in another profile. Additional research would be needed for this.

We have added a short comment about this to the Discussion:

Page 14, line 498-504 (in revised manuscript with track changes):

‘For example, by having students focus on a specific task performance within a game

situation or by working in same skill level groups, in which social comparison is avoided

as much as possible. However, in order to determine if students in a given profile may

thrive better in a specific learning climate (i.e. performance or mastery climate) or benefit

more from, for example, providing choices during learning tasks (i.e. autonomy support)

than students in another profile, further intervention studies would be needed.’

Our results (table 5) do show that in all three profiles, mastery approach goals scores

higher than performance approach goals, while performance avoidance goals scores

higher than mastery avoidance goals. This would argue against the suggestion that a

performance climate may be more beneficial for some students.

**Comments reviewer**

**1. *Great introduction and reason to conduct the study. Good explanations of***

***SDT and AGT. Very detailed methods section and results section. The***

***graphs in the results section were very understandable and went along with***

***the descriptions.***

Thank you for your compliments and clear comments**.**

***2. The one thing that I would state as a limitation would be the measurement***

***for leisure time sport participation. Is regular physical activity considered***

***sport participation in the Netherlands? I feel students might find it***

***confusing and might not answer accurately on this type of questionnaire.***

Thank you for this comment. It made us aware of the fact that we insufficiently take into

account the cultural and contextual differences between countries and the need to

provide more background information.

In general, regular physical activity is not considered as sport participation in the

Netherlands. For example, given the short distances and the flatness of our country

riding bicycles is considered as a normal part of life and not as sport participation.

The Netherlands also have a strong system and tradition of voluntary sports clubs. With

62 sports clubs on average per municipality (11 sports clubs per 10.000 inhabitants),

sports clubs play a central role in the Dutch sports system. The Netherlands have a high

rate of participation in sports and ranks highest in the European Union in terms of

membership of sports clubs (Pulles & Breedveld, 2014).

From this perspective we asked students for their leisure time sport participation. In

addition, in the original questionnaire we included some examples of leisure time sport

participation activities to make this even more clear to the respondents, but we did not

add these to the first draft of this paper. Therefore, in the revised manuscript we now

added some more information:

Page 8, line 306-313 (in revised manuscript with track changes):

*‘Leisure Time Sport Participation (LTSP).* The Netherlands have a strong system

and tradition of sports clubs. With 62 sports clubs on average per municipality,

sports clubs play a central role in the Dutch sports system. The self-reported

amount of students’ sport participation during leisure time was measured using the

single item *‘Do you practice sports/ sports activities outside the school setting?*

*(for example, football, field hockey, tennis, fitness, swimming, dancing,*

*gymnastics, etc.)*’. The response options were (1) ‘Less than 1 time a week on

average’; (2) 1 or 2 times a week on average; (3) ‘3 or more times a week on

average’.’

**References**

Haerens, L., Aelterman, N., Van den Berghe, L., De Meyer, J., Soenens, B., &

Vansteenkiste, M. (2013). Observing physical education teachers' need-supportive

interactions in classroom settings. *Journal of Sport & Exercise Psychology, 35(1),* 3–17.

Morgan, K., Sproule, J., Weigand, D., & Carpenter, P. (2005). A computer-based

observational assessment of the teaching behaviours that influence motivational climate

in Physical Education. *Physical Education & Sport Pedagogy, 10(1)*, 83–105.

Pulles, I., & Breedveld, K. (2014). *NL a sporting nation: A brief glance into Dutch sports,*

*factsheet.* Utrecht, The Netherlands: Mulier Institute.
